# Youth green aesthetic perception and psychological influence on the adoption of sustainable packaging for biomimetic cultural products in social media environments

**DOI:** 10.3389/fpsyg.2026.1752048

**Published:** 2026-04-14

**Authors:** Tingyun Luo, Juiche Tu, Lixia Liu, Yuan Miao, Ting Liu

**Affiliations:** 1 School of Design, Fujian University of Technology, Fuzhou, China; 2 Department of Creative Design, National Yunlin University of Science and Technology, Yunlin, Taiwan; 3 College of Art, Beijing Union University, Beijing, China

**Keywords:** biomimetic design, cultural and creative products, green packaging, social media, youth green aesthetics

## Abstract

**Introduction:**

Driven by global carbon reduction agendas and circular economy initiatives, green packaging has evolved beyond regulatory compliance toward value creation and identity expression. In social media environments, young consumers assess and disseminate products through intertwined considerations of sustainability, aesthetics, and social interaction. Within this context, biomimetic cultural and creative products offer a unique lens to explore how youth green aesthetics influence the adoption of green packaging. Drawing on innovation diffusion theory, persuasive design theory, and biophilia theory, this study develops an integrated analytical framework to examine the underlying psychological and behavioral mechanisms.

**Methods:**

A cross-sectional research design was employed, combining qualitative expert interviews with quantitative survey analysis. Data were collected from 662 young respondents across mainland China and Taiwan. Structural equation modeling (SEM) was used to test the proposed relationships, including mediation effects of perceived innovation attributes (relative advantage, compatibility, observability), persuasive design factors (motivation, ability, triggers), and biophilic responses (natural imagery evocation and biomimetic experience).

**Results:**

The findings indicate that youth green aesthetics have a significant positive effect on the willingness to adopt green packaging. This relationship is mediated by perceived innovation attributes, persuasive design mechanisms, and biophilic responses. Among these mediators, biomimetic experience and perceived ability demonstrate the strongest effects, while observability does not exhibit a significant mediating role. The results highlight the joint influence of aesthetic perception, behavioral facilitation, and nature-related emotional engagement in shaping adoption intentions.

**Discussion:**

Rather than establishing causal claims, this study provides empirical evidence of the mechanisms through which youth green aesthetics influence green packaging adoption within social media contexts. The integration of diffusion, persuasion, and biophilia perspectives offers a novel theoretical contribution to sustainable design research. Practically, the findings inform green packaging design strategies and sustainability communication in the cultural and creative industry. Future research is encouraged to adopt longitudinal and cross-cultural approaches to further validate and extend these findings.

## 1 Introduction

With the advancement of global carbon reduction governance and the circular economy agenda, green packaging has gradually shifted from a form of regulatory compliance toward a medium of value co-creation, cultural expression, and identity signaling. This transformation is particularly evident within social media environments, where short-video platforms, live-streaming e-commerce, and online communities have become central arenas for product evaluation, reinterpretation, and dissemination. Within these contexts, young consumers increasingly engage with green packaging through an integrated logic of sustainability, aesthetics, and social interaction (Adriaens, 2019).

At the same time, innovations inspired by biomimicry in form, structure, and materials have expanded the meaning of green packaging beyond functional and environmental performance. Bionic cultural and creative packaging embeds natural metaphors, tactile experiences, and cultural narratives, transforming packaging into a visual and emotional interface between ecological values and everyday consumption (Deng and Yang, 2024). While existing studies have examined green packaging from perspectives such as functionality, recyclability, or environmental attitudes, they remain fragmented and largely descriptive. In particular, there is a lack of integrative empirical models that explain how youth green aesthetics translate into adoption behavior through social media visualization and diffusion mechanisms, and why aesthetic engagement can be transformed into sustained pro-environmental action (Aguilar-Planet and Peralta, 2024; Badarnah and Kadri, 2015).

To address this gap, this study proposes an integrated theoretical framework that combines three diffusion perspectives with persuasive design theory and biophilia theory. Rather than treating these theories as parallel explanations, the framework assigns each a distinct analytical role across micro-, meso-, and macro-levels of adoption. This positioning clarifies their complementarity while avoiding conceptual redundancy.

At the micro level, the study draws on Rogers’ Diffusion of Innovations Theory to examine how perceived innovation attributes—relative advantage, compatibility, complexity, trialability, and observability—shape individual adoption decisions. In social media contexts, observability is substantially enhanced through image-based sharing, unboxing videos, and influencer demonstrations, while trialability is supported by experiential content such as AR simulations and low-threshold usage scenarios. Importantly, youth green aesthetics are conceptualized not as an outcome variable, but as an antecedent value orientation that shapes how these innovation attributes are perceived. In this sense, green aesthetics function as a value-based cognitive lens that amplifies perceived compatibility with lifestyle and identity, as well as the symbolic relative advantage of environmentally expressive packaging (Forniés and Muro, 2010; Yuan and Xing, 2019).

At the meso level, diffusion is examined through social influence mechanisms such as opinion leadership, two-step flow communication, and social contagion. Social media creators and key opinion leaders translate green aesthetics into stylized narratives using bionic metaphors, material storytelling, and cultural symbolism, while simultaneously embedding social identity cues such as environmental badges or carbon-reduction indicators. These aesthetic expressions operate as visual signals of value homophily and group belonging, generating normative pressure and reputational incentives for imitation within peer networks (Wu and Chen, 2015; Gosal et al., 2024). Here, green aesthetics do not merely reflect personal preference but serve as socially legible markers that facilitate collective diffusion (Mahmoud et al., 2022).

At the macro level, the Bass diffusion model is employed to explain how external influences (p), such as platform algorithms, brand campaigns, and media exposure, interact with internal influences (q), including peer interaction and user-generated content. Social media ecosystems intensify both parameters by algorithmically amplifying visually appealing green packaging content while enabling rapid imitation within interest-based communities. When bionic green aesthetics are repeatedly reinforced by core influencers, an “aesthetic paradigm” emerges, increasing the elasticity of the imitation coefficient and accelerating the S-shaped adoption trajectory among youth consumers (Lenau, 2017; Xiao and Seong, 2025).

Building upon this three-layer diffusion structure, persuasive design theory is introduced to explain the behavioral transition from aesthetic identification to concrete action. Drawing on the Fogg Behavior Model, this study distinguishes motivation, ability, and trigger as functionally distinct components (Letard et al., 2025). Green aesthetics primarily contribute to motivation by fostering value resonance and affective attraction, while design features such as intuitive opening mechanisms, clear recyclability cues, and pleasant tactile feedback enhance perceived ability. Triggers are operationalized through persuasive cues embedded in both platforms and packaging, including real-time carbon feedback, recycling incentives, ritualized unboxing prompts, and social proof mechanisms (Azizkhani, 2015). This clarification addresses potential overlap between motivation and emotional outcomes by positioning persuasive design as the mechanism that activates behavior rather than as an affective construct itself (Liu et al., 2022).

Finally, biophilia theory provides the psychological foundation underlying aesthetic motivation by explaining why natural forms and textures elicit emotional engagement in the first place (Wadia and McAdams, 2010; Yuxin et al., 2025). Biophilic elements such as vein-like patterns, honeycomb structures, natural color palettes, and fiber-based textures evoke innate human affinity toward nature, generating emotional restoration, moral elevation, and a sense of ethical satisfaction or “warm glow” (Perez et al., 2011). In this framework, biophilic experience is treated as the source of emotional fulfillment rather than as a behavioral driver, thereby reducing conceptual overlap with motivation constructs. This emotional grounding reinforces perceived innovation advantages and supports the formation of immersive aesthetic paradigms that are easily imitated within social diffusion processes (Glier et al., 2011).

Through this theoretically differentiated integration, the proposed framework offers a coherent explanation of how youth green aesthetics function simultaneously as a value orientation, an emotional catalyst, and a social signal, collectively shaping the adoption of green packaging for biomimetic cultural and creative products in social media environments.

### 1.1 Diffusion of innovations theory

Rogers’ innovation diffusion theory (1962/2003) highlights that the adoption of innovations within social systems is shaped by communication channels, time, social structure, and individual characteristics (de Sá and Viana, 2023; Razzak and De Brabander, 2011). This theory offers a logical framework for understanding how young consumers transition from early adopters to the mainstream in the realm of green packaging and bionic cultural and creative products. Studies indicate that the adoption of green packaging, as a novel sustainable design strategy, frequently hinges on five key attributes: relative advantage, compatibility, complexity, trialability, and observability (Rácz et al., 2013; Seifollahi, 2023).

In the realm of social media, the dynamics of innovation diffusion are intensified by algorithmic recommendations and online community interactions (Winkler, 2017). For example, the spread of green aesthetics and bionic packaging design concepts through short videos, user-generated content comments, and key opinion leader endorsements boosts the visibility and credibility of these innovations, thereby speeding up their diffusion. Additionally, the identity formation and group imitation tendencies among young people make green aesthetics not just a consumer choice but also a social expression of personal values (George et al., 2023). The literature suggests that research in this area should integrate interdisciplinary analysis of digital scenario variables and aesthetic values to more fully uncover the psychological and social mechanisms behind the adoption of green packaging (George, 2025).

### 1.2 Persuasive design theory

Fogg’s persuasive design theory, introduced in 2003, focuses on altering individuals’ attitudes and behaviors through systematic design rather than external coercion. The Fogg Behavior Model (FBM) asserts that behavior occurs when three elements—motivation, ability, and trigger—are present simultaneously (Chen and Hunt, 2007; Wommer and Wanieck, 2022). In the realm of green packaging, young consumers’ green aesthetic preferences act as motivation drivers. Simplifying the user experience, such as providing easy opening and intuitive recycling prompts, enhances behavioral ability. Meanwhile, interactive reminders on social media, like green labels, challenges, and trending environmental topics, serve as triggers (Gertik and Karaman, 2023; Granero, 2019; Luo et al., 2024).

Research indicates that persuasive design in green consumption behavior has developed into three distinct paths. The first is the symbolic path, which evokes resonance with green values through visual symbols and aesthetic coding. The second is the interactive path, which reduces adoption barriers via user-friendly interfaces and information feedback. The third is the social path, which promotes sustainable behavior through group identity and social comparison. On social media, these paths intertwine, forming a cyclic process centered on “green aesthetic symbols - behavior triggering mechanism - community identity feedback” (Romlee et al., 2016). In ongoing social interactions, young users view adopting green packaging as a “socially visible aesthetic behavior,” rather than just a functional choice (Chen and Chen, 2014). This perspective transcends traditional persuasion theory, highlighting the dynamic interplay between aesthetics and behavior transformation in digital media contexts.

### 1.3 Biophilia theory

The biophilia theory, introduced by Wilson in 1984, suggests that humans have an inherent affinity for and psychological reliance on natural elements, developed through evolution. In studies on green packaging and bionic cultural and creative products, this theory helps explain why natural textures, ecological symbols, and bionic designs resonate aesthetically and emotionally with young consumers (Caldera et al., 2021; Terrier et al., 2019; Yu, 2023). Research indicates that packaging designs featuring natural material textures (like pulp and bamboo fiber), bionic textures (such as vein structures and shell spirals), and ecological colors (like natural green and soil brown) can evoke emotional responses and environmental awareness in consumers, thereby enhancing their positive attitudes toward green consumption.

In social media, biophilic design not only satisfies young people’s psychological desire to “return to nature” through image sharing but also complements green aesthetics (Stroble et al., 2009). Young people often create a cultural identity centered on “sustainable lifestyles” during virtual interactions, with biophilic design serving as a tangible visual symbol (Drack et al., 2018). Recent literature highlights the dual value of biophilic theory in green packaging research: it provides emotional restoration (restorative effect) and fosters environmental responsibility and a willingness for collective action (pro-environmental behavior) (Roth-Nebelsick and Krause, 2023). Thus, integrating biophilic theory with green aesthetics can more profoundly explain why young people exhibit high sensitivity and proactive adoption of green packaging in the social media context.

### 1.4 Research description

To further clarify the conceptual integration of the three theoretical frameworks, the present study distinguishes the functional roles of diffusion theory, persuasive design theory, and biophilia theory within the analytical model. Although some constructs—particularly motivation and emotional fulfillment—may appear conceptually related, they represent different stages and mechanisms within the adoption process.

First, innovation diffusion theory explains the cognitive evaluation stage of adoption. In this study, constructs such as relative advantage, compatibility, and observability represent individuals’ perceptions of innovation attributes. These factors determine whether green packaging is interpreted as beneficial, socially compatible, and worth adopting. Diffusion theory therefore operates primarily at the level of perceived innovation value and social transmission mechanisms.

Second, persuasive design theory, based on the Fogg Behavior Model, focuses on the behavioral activation stage. The constructs of motivation, ability, and trigger represent functional conditions that enable the translation of positive perceptions into actionable intention. Within this framework, motivation refers to goal-oriented behavioral readiness, while ability and trigger reduce practical barriers and activate the decision process. Persuasive design thus explains how favorable perceptions are converted into behavioral intention.

Third, biophilia theory provides the affective and psychological foundation underlying aesthetic engagement with nature-inspired design. Constructs such as natural imagery, biomimetic experience, and emotional fulfillment capture the emotional resonance elicited by natural forms, materials, and ecological symbolism. Importantly, emotional fulfillment in this study reflects affective responses toward nature-related design, rather than behavioral motivation itself.

Consequently, while motivation and emotional fulfillment both relate to positive internal states, they operate at different analytical levels. Emotional fulfillment represents an affective response generated by biophilic stimuli, whereas motivation represents a behavioral readiness activated through persuasive mechanisms. This conceptual distinction allows the three theoretical perspectives to complement one another rather than overlap, forming a multi-layer explanatory framework linking aesthetic perception, emotional resonance, and behavioral activation in the adoption of green packaging.

Based on the above integration, this paper presents the following core research questions:

(1) How does youth green aesthetics influence the intention and behavior of adopting green packaging for biomimetic cultural and creative products through perceived innovation attributes (Rogers)?
(2) How do opinion leaders and peer interactions on social media function as mediating or moderating mechanisms that amplify the diffusion effect of green aesthetics (two-step flow and social contagion)?
(3) How do persuasive design elements—motivation, ability, and trigger, along with specific strategies—affect the transformation efficiency from “aesthetic perception” to “adoption,” and how do they interact with perceived innovation attributes?

**Figure 1:**
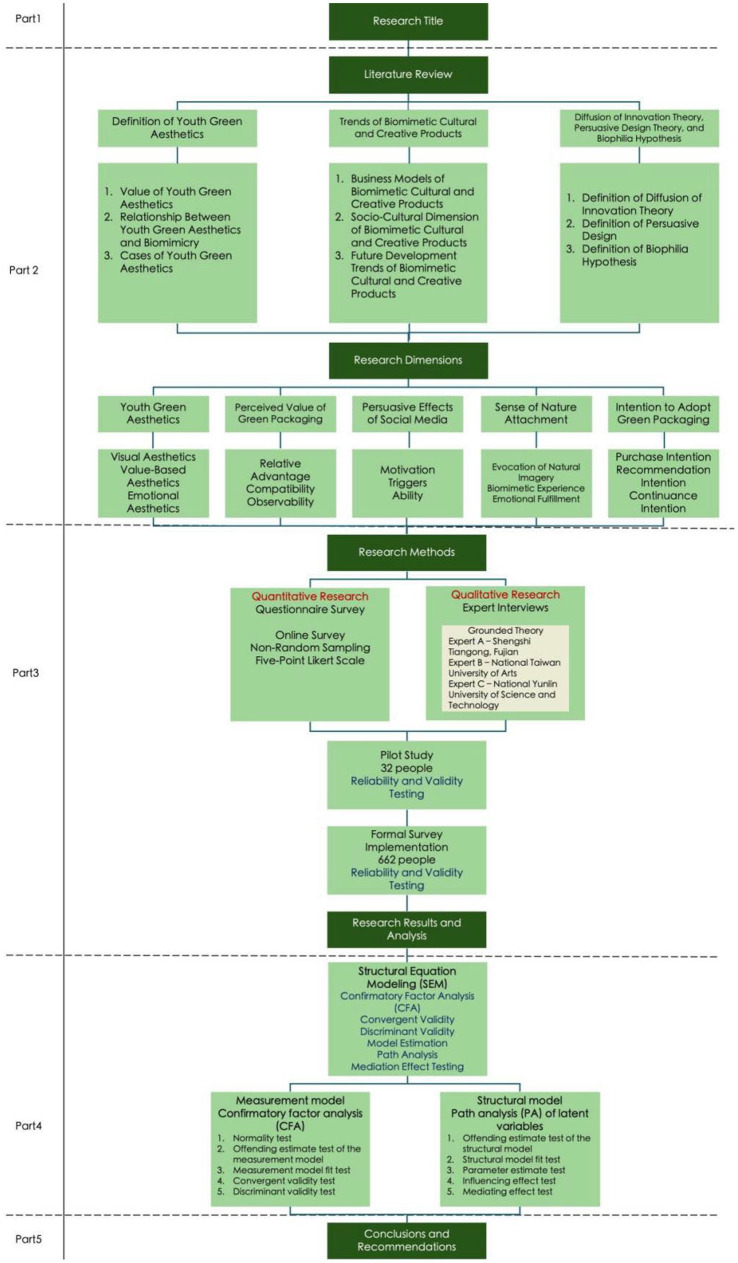
Research framework diagram.

The research framework diagram is shown in Figure 1:

This paper makes three key theoretical contributions. First, it introduces a cross-level integrated framework of “aesthetics–diffusion–persuasion–biophilia” to address the gap in coupling aesthetic factors with diffusion dynamics in green packaging research. Second, it embeds a persuasion design mechanism within the innovation diffusion process to demonstrate how social media micro-interactions lower adoption thresholds and enhance conversion efficiency. Third, it applies biophilia theory to elucidate the unique emotional and ethical benefits of biomimetic packaging, clarifying how natural imagery serves as the “content atom” for visual and replicable social diffusion, thereby linking micro-psychology with the macro-diffusion curve.

## 2 Research methods

### 2.1 Expert interview method

This study’s expert interviews involved the CEO of Fujian Shengshi Tiangong, a professor from the Institute of Creative Industries at Taiwan University of the Arts, and a professor from the Department of Creative Life at Yunlin University of Science and Technology in Taiwan. The research utilized 30-min semi-structured interviews, informed by in-depth discussions and expert suggestions, to gather professional insights and information. The aim was to understand the development trends and context of young people’s green aesthetics in the packaging of bionic cultural and creative products. This understanding will serve as a reference for identifying future key and strategic factors.

To ensure the conceptual validity of the questionnaire items, an expert interview phase was conducted prior to the large-scale survey. Three experts were purposively selected based on their professional expertise in design practice, cultural and creative industries, and sustainable design research. The selection criteria included: (1) more than 10 years of professional experience in design or cultural and creative industries, (2) recognized academic or industrial contributions related to product design, green design, or cultural creativity, and (3) familiarity with emerging trends in sustainable packaging or biomimetic design. The inclusion of experts from both academia and industry was intended to ensure a balanced perspective that integrates theoretical insight with practical experience.

Semi-structured interviews lasting approximately 30 min were conducted online. The interview protocol consisted of five thematic dimensions—conceptual understanding, social influence, developmental change, marketing considerations, and future trends—designed to explore how youth green aesthetics and biomimetic design are interpreted in contemporary packaging contexts. The semi-structured format allowed the researchers to maintain a consistent interview structure while enabling experts to elaborate on emerging themes and provide practical insights.

The interview data were analyzed using a qualitative coding procedure inspired by grounded theory. First, open coding was applied to identify recurring concepts related to youth green aesthetics, social media influence, sustainable packaging values, and biomimetic experience. Second, axial coding was conducted to group these concepts into broader thematic categories, including aesthetic cognition, social diffusion mechanisms, consumer decision processes, and future development expectations. Through iterative comparison and discussion among the research team, the coding results were synthesized into a structured set of conceptual dimensions.

The insights derived from this qualitative analysis directly informed the development of the questionnaire. Specifically, the themes identified during coding were translated into measurable constructs and item statements corresponding to the theoretical variables used in the study, including youth green aesthetics, perceived innovation attributes, persuasive design mechanisms, biophilic responses, and adoption intention. This process ensured that the questionnaire items were not only grounded in existing literature but also reflected contemporary industry practices and expert knowledge.

To further enhance content validity, the preliminary questionnaire was reviewed by the same experts, who provided feedback regarding wording clarity, conceptual relevance, and measurement completeness. Minor revisions were made accordingly before the questionnaire was administered in the pilot test and subsequent large-scale survey. Details of the expert interview subjects are presented in Table 1.

**Table 1:** Respondents of expert interviews.

Code	Interviewee	Title	Affiliation	Relevant Experience / Research Areas	Interview Date / Method
A	Huang◯◯	CEO	Shengshi Tiangong, Fujian	Product design, industrial design, engineering design, marketing, design planning and management	2025.09.13 Online
B	Lin◯◯	Professor	Graduate Institute of Creative Industries, National Taiwan University of Arts	Interdisciplinary integration of cultural and creative industries, creative design, human factors engineering, product marketing, design management	2025.09.13 Online
C	Du◯◯	Professor	Department of Creative Design, National Yunlin University of Science and Technology	Green design, lifestyle design, industrial design, design education, cultural and creative design	2025.09.14 Online

The interview outline is organized into five main categories: (1) Conceptual aspect, (2) Social aspect, (3) Development aspect, (4) Sales aspect, and (5) Trend aspect. By engaging in discussions with experts and scholars, grounded theory is utilized to distill key elements within each category, providing a foundation for designing the questionnaire items in this study. Table 2 displays the items included in the expert interviews.

**Table 2:** Items for expert interviews.

Dimension	Item code	Interview question
A. Conceptual dimension	A-1	How do you define youth green aesthetics? In what ways does it differ from conventional “beauty” or “fashion” aesthetics?
	A-2	In your view, what is the relationship between biomimetic cultural and creative products and green packaging?
	A-3	What specific actions or behaviors does adopting green packaging represent to you?
B. Social dimension	B-1	On social media, which people or communities most influence your perceptions and choices regarding green packaging?
	B-2	When discussions arise within your circle about whether “green packaging is essential or unnecessary,” how do you respond?
	B-3	Do you regard choosing green packaging as a form of self-expression or a personal identity symbol?
C. Developmental dimension	C-1	Looking back over the past 1–2 years, how have your attitudes and behaviors toward green packaging changed?
	C-2	Please recall a complete experience from being influenced or inspired (“seeded”) to actually purchasing or using a green-packaged product.
	C-3	How do your interactions with brands/designers/sellers (e.g., comment sections, product testing, co-branding, or co-creation) influence your understanding and trust of green packaging?
D. Marketing dimension	D-1	When similar products with green packaging are priced higher, what premium range are you willing to accept, and why?
	D-2	In content or live-commerce scenarios, what types of presentation most effectively motivate you to purchase green-packaged biomimetic cultural products?
	D-3	How do after-sales experiences and secondary sharing (e.g., posting reviews, recycling incentives) influence your repurchase intention and word-of-mouth recommendations?
E. Trend dimension	E-1	What kinds of “more natural” or “smarter” innovations do you expect to see in future green packaging for biomimetic cultural products?
	E-2	How do you perceive the role of policies and standards (e.g., plastic reduction, recycling labels, carbon labeling) in promoting or constraining the adoption of green packaging?
	E-3	Looking ahead 2–3 years, what changes in the social media ecosystem are most likely to reshape your attitudes and behaviors toward green packaging?

### 2.2 Structural equation model

Structural equation modeling (SEM) is a comprehensive network that integrates analytical techniques, including mathematical models, computer algorithms, and statistical methods. This multivariate statistical approach combines factor analysis and path analysis, primarily focusing on quantitatively examining the interactive relationships among multiple variables. Over the past three decades, SEM has been widely utilized in social and behavioral sciences and has recently gained traction in consumer market research.

Structural equation models are primarily categorized into two types: the confirmatory factor analysis diagram, also referred to as the measurement model diagram, and the latent variable path analysis diagram, also known as the structural model diagram. These models incorporate two types of variables. The first type is the structural variable, an unobservable variable also called the latent variable, depicted by an oval. The second type is the observed variable, which can be obtained through methods like questionnaires or interviews and is represented by a rectangle.

Although the proposed research framework integrates innovation diffusion theory, persuasive design theory, and biophilia theory into a coherent cross-level structure, structural equation modeling (SEM) requires explicitly stated, directional hypotheses to ensure methodological rigor and transparency. To address this concern, the present study formulates a set of theoretically grounded hypotheses that correspond one-to-one with the structural paths tested in the SEM model. This clarification allows the framework to be evaluated as a theory-driven explanatory model rather than an exploratory configuration.

#### 2.2.1 Research hypotheses

This study employs constructs such as youth green aesthetics, perceived value of green packaging, persuasion effect of social media, natural attachment, and green packaging adoption intention, derived from a comprehensive compilation and analysis of literature reviews. Building on theoretical models developed by previous scholars, the study formulates research hypotheses aimed at confirming causal relationships among these factors. These hypotheses are designed to elucidate the influences and effects among the constructs, thereby providing a foundation for further research analysis and validation.

The hypotheses of this study are as follows:

1 Direct effect

- H1: The youthful green aesthetic significantly enhances the willingness to adopt eco-friendly packaging.

2 Mediating effect: Diffusion of Innovations theory

- H2a: The green aesthetics appealing to youth positively impact their intention to adopt green packaging by highlighting its relative advantage.
- H2b: The green aesthetic preferences of young people positively influence their willingness to adopt green packaging by enhancing compatibility.
- H2c: The green aesthetic appeal to youth positively influences their willingness to adopt green packaging by enhancing its observability.

3 Mediating effect: Persuasive Design Theory

- H3a: The green aesthetic preferences of young people positively influence their motivation to adopt green packaging.
- H3b: The green aesthetic preferences of young people positively influence their willingness to adopt green packaging when activated by specific triggers.
- H3c: Young people’s ability to perceive green aesthetics positively influences their willingness to adopt green packaging.

4 Mediating effect: Biophilia theory

- H4a: The green aesthetics appreciated by youth positively impact their willingness to adopt green packaging, facilitated by natural image
- H4b: The green aesthetics of young people positively influence their willingness to adopt green packaging through bionic experience.
- H4c: The green aesthetic appeal to youth positively influences their willingness to adopt green packaging by providing emotional satisfaction.

Together, these hypotheses establish a clear one-to-one correspondence between theoretical constructs and the structural paths tested in the SEM model. This explicit hypothesis specification enhances methodological transparency, clarifies the study’s theory-testing orientation, and allows for a rigorous assessment of both supported and unsupported theoretical expectations.

#### 2.2.2 Questionnaire design

This study focused on young adults in China, specifically those aged 18 and older. We employed a non-random sampling method using an online Likert scale questionnaire. Items C06 and D05 were reverse-scored and adjusted during statistical analysis. Initially, the questionnaire underwent pre-testing with a sample of 32 participants. We then assessed its reliability and validity. Items failing to meet the required standards were removed before distributing the final version of the questionnaire. According to the ratio of estimated parameters to sample size in the maximum likelihood estimation method should be at least 1:10. To ensure the data’s reliability and validity, we distributed a total of 662 questionnaires, with 332 completed by males and 330 by females.

In this study, we utilized IBM SPSS 26 and IBM AOMS 25 for structural equation model analysis. By examining the interactions and relationships among service quality, perceived value, and consumers’ willingness to use naked shops, we identified key elements and criteria for future marketing strategies of natural information products. Table 3 presents the design of the questionnaire items.

**Table 3:** Key elements of bionic cultural and creative product packaging.

No.	Dimension	Key elements
1	Conceptual cognition and value positioning	(1) Uniqueness of youth green aesthetics Compared with conventional “beauty” or “fashion” aesthetics, youth green aesthetics focuses more on the comprehensive expression of naturalness, environmental responsibility, and moral consciousness. It has become an important symbol for young people to differentiate themselves from traditional consumer culture. (2) Symbiotic relationship between biomimetic cultural products and green packaging Biomimetic cultural and creative products pursue a “learning-from-nature” design logic, while green packaging represents an extension of that ecological value. Their integration reinforces the concept of “ecological integrity,” achieving coherence in functionality, visuality, and ethics. (3) Behavioral connotation of green packaging adoption “Adoption” goes beyond simple purchasing decisions; it encompasses the entire consumption chain, including actively seeking eco-labeled products, supporting sustainable brands, and participating in recycling initiatives. This process signifies both a consumer choice and a form of social responsibility practice.
2	Social interaction and identity construction	(1) Influence of social media communities Opinion leaders, lifestyle influencers, and eco-communities on platforms such as Xiaohongshu and Weibo exert substantial influence on youths’ attitudes toward green packaging. Peer groups and micro-communities often drive group-based imitation and diffusion effects. (2) Value expression in circle discussions When encountering polarizing debates such as “green packaging is essential vs. redundant,” young consumers tend to take a stand. Choosing green packaging becomes not only a consumption decision but also a way to express civic awareness and social participation. (3) Symbolic identity of green packaging For young people, adopting green packaging serves as an “identity label.” It represents recognition of sustainable culture and becomes a narrative resource for displaying one’s lifestyle on social media.
3	Developmental change and consumer journey	(1) Evolution of attitudes and behaviors over time Experts note that in the past 1–2 years, youths’ cognition toward green packaging has evolved from “optional” to “expected.” This shift is closely linked to the increasing social visibility of environmental topics, corporate transparency, and policy guidance. (2) Mechanism from “seeding” to “ordering” The purchasing process among young consumers often unfolds within the “narrative context” of social media—from influencer recommendations to comment interactions and final purchasing experiences—where green packaging acts as a trust- and affinity-enhancing cue. (3) Brand interaction and trust building Youths’ understanding and trust in green packaging are shaped by brand engagement through comment responses, co-branded campaigns, and transparent communication. Such interactions strengthen consumer perception of corporate authenticity and environmental commitment.
4	Consumer acceptance and purchasing decisions	(1) Reasonable range of price premium tolerance While young consumers generally accept price premiums for green packaging, the range remains moderate. Most experts indicate that a 5–15% premium is acceptable, justified by the added environmental and social value perceived to offset price sensitivity. (2) Trigger effects of social media and live-commerce scenarios The most effective purchasing triggers arise from lifestyle representation and alignment with environmental values. Visual storytelling with natural imagery and authentic influencer testimonials significantly increase adoption intent. (3) Repurchase motivation from post-purchase and secondary sharing Post-consumption sharing behaviors (e.g., unboxing posts, recycling rewards, and online reviews) reinforce consumer-brand relationships and generate word-of-mouth diffusion, serving as vital drivers for sustained adoption of green packaging.
5	Future trends and policy drivers	(1) Innovation expectations for green packaging Future green packaging innovations are expected to combine “more natural” and “more intelligent” features—such as bio-based degradable materials, circular design with interactive reuse, and packages with educational or artistic attributes. (2) Dual effects of policies and standards Governmental policies (e.g., plastic reduction directives, recycling symbols, and carbon labeling) provide systemic assurance for green packaging adoption. Standards function both as constraints and incentives, enhancing transparency, curbing “greenwashing,” and increasing consumer trust. (3) Reshaping potential of social media ecosystems In the next 2–3 years, the evolving digital ecosystem will amplify green discourse: platform algorithms will prioritize sustainability topics, and virtual communities will strengthen eco-identity clustering, further reshaping youths’ attitudes and behaviors toward green packaging.

To ensure transparency in the data collection process, the recruitment procedure and distribution channels of the questionnaire are clarified as follows. The survey was conducted using a non-probability online sampling strategy targeting young social media users who have experience interacting with cultural and creative products or sustainability-related content.

The questionnaire was distributed through several widely used social media and online communication platforms in Mainland China and Taiwan, including WeChat groups, Xiaohongshu communities, and online student forums, as well as university alumni networks and design-related social media groups. These channels were selected because they represent common environments where young consumers exchange information about cultural products, sustainability topics, and lifestyle trends.

Participation in the survey was voluntary and anonymous. Before answering the questionnaire, respondents were informed about the academic purpose of the study and were asked to confirm that they were 18 years of age or older. No personally identifiable information was collected, ensuring confidentiality and ethical compliance.

To enhance data quality, several data screening procedures were applied. First, responses with incomplete questionnaires or missing values were removed. Second, responses showing extremely short completion times or clear response patterns (e.g., identical answers across all items) were excluded. Third, reverse-scored items were used to detect inattentive responses. After data cleaning, a total of 662 valid questionnaires were retained for statistical analysis.

Although the sampling method was non-random, the respondents were drawn from multiple social media communities across Mainland China and Taiwan, providing a diverse sample of young users who are active in digital cultural consumption environments. This sampling strategy is consistent with prior studies investigating social-media-driven consumer behavior and sustainability adoption.

### 2.3 Data visualization analysis

#### 2.3.1 Keyword co-occurrence analysis

**Figure 2:**
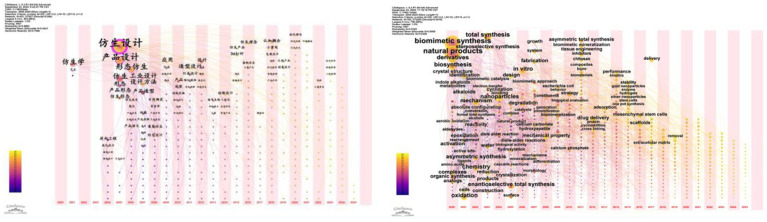
Time zone map with Chinese and English keywords.

**Figure 3:**
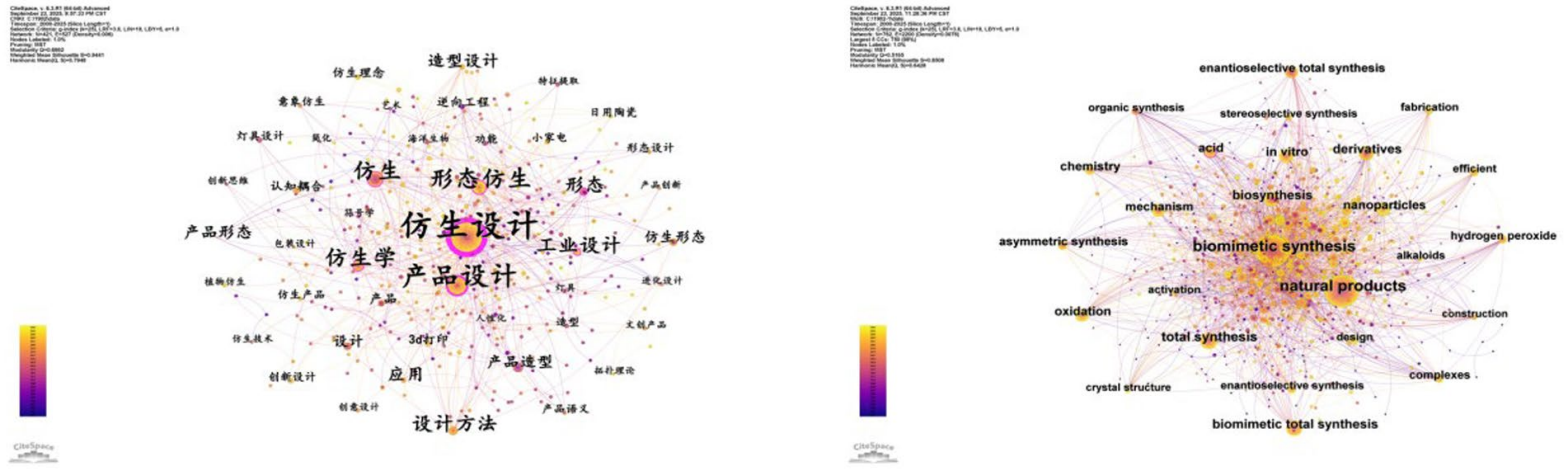
Co-occurrence map of Chinese and English keywords.

In this study, we utilized CiteSpace software to analyze keywords from research literature on bionic products in the CNKI and WOS databases. This analysis produced keyword co-occurrence maps for both Chinese and English literature, as illustrated in Figure 2. In the CNKI database, “bionic design” appeared most frequently, with 167 occurrences and a centrality of 0.89. Meanwhile, in the WOS Core Collection database, “biomimetic synthesis” topped the list with 626 occurrences and a centrality of 0.06, as depicted in Figure 3 and Table 4.

**Table 4:** Top 10 keywords in Chinese and English literatures.

No.	CNKI Database			No.	WOS Core Database		
	Frequency	Frequency	Centrality		Frequency	Frequency	Centrality
1	仿生设计	167	0.89	1	Biomimetic synthesis	626	0.06
2	产品设计	68	0.37	2	Natural products	561	0.02
3	形态仿生	34	0.12	3	Total synthesis	213	0.06
4	仿生	33	0.16	4	Biomimetic total synthesis	181	0.07
5	仿生学	29	0.16	5	Derivatives	180	0.08
6	工业设计	19	0.13	6	Biosynthesis	168	0.04
7	设计方法	18	0.08	7	Nanoparticles	155	0.04
8	形态	15	0.11	8	Chemistry	148	0.07
9	应用	12	0.04	9	Oxidation	144	0.08
10	造型设计	11	0.07	10	*In vitro*	144	0.06

In the CNKI database, “product design” and “morphological bionics” appear with frequencies of 68 and 34, respectively. Conversely, in the WOS database, “natural products” and “total synthesis” are prominent, with frequencies of 561 and 213, highlighting the global research focus on these topics. Furthermore, in the CNKI database, “bionics” and “industrial design” exhibit high centralities of 0.16 and 0.13, respectively, indicating their strong connections with other keywords in Chinese literature and their importance as key nodes in the research network. In the WOS database, “biomimetic total synthesis” and “derivatives” show centralities of 0.07 and 0.08, respectively, underscoring their critical roles in shaping the knowledge network within the field of biomimetic products.

The data indicate that keyword co-occurrence maps across various databases highlight the diversity and complexity of research in bionic products. Chinese literature tends to emphasize bionic design and product form, whereas international literature focuses more on bionic synthesis and natural products. These variations may arise from differing research traditions, cultural backgrounds, and academic priorities. Future research could delve deeper into the reasons for these disparities and aim to identify the universal principles of bionic products and their applications within specific contexts in a globalized setting.

#### 2.3.2 Analysis of research frontiers

**Figure 4:**
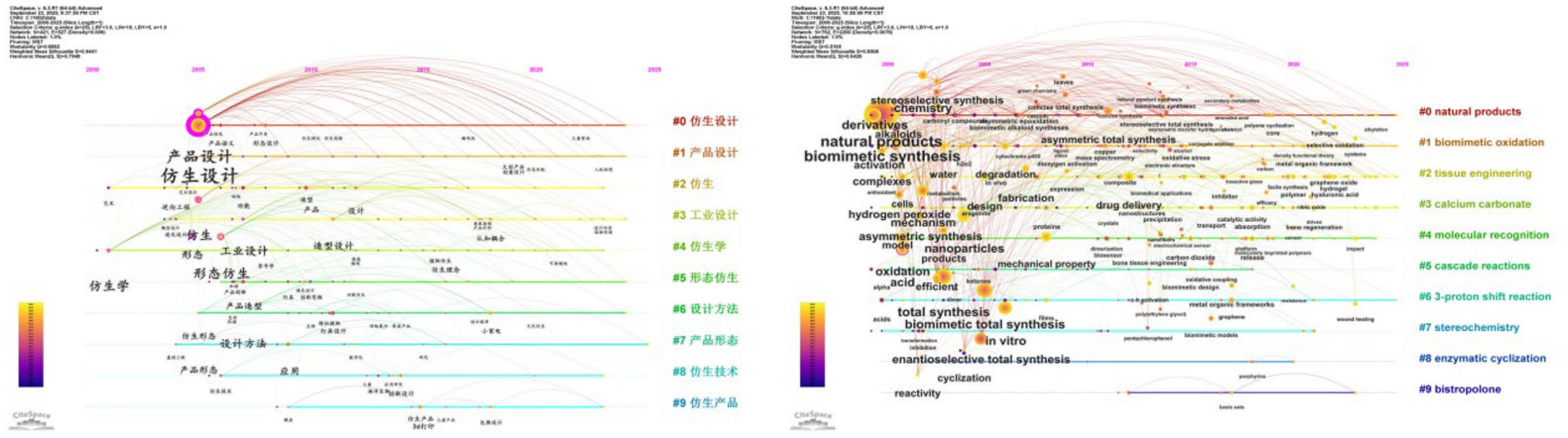
Timeline of Chinese and English keywords.

The timeline map of keyword clustering uses the vertical axis for cluster name labels and the horizontal axis for publication years. A node’s position indicates the first appearance of the corresponding keyword. By clustering the keyword network, a frontier timeline map is generated, as depicted in Figure 4. In this map, each circle symbolizes a keyword, placed in the year it first appeared. Furthermore, burst analysis is performed on the top 15 keywords by research ranking to trace the development history and research frontiers in this field, resulting in the keyword burst information map shown in Figure 2. The following provides a comparative analysis of research trends in this area:

1 Initial stage: During the initial stage, both Chinese and English literature concentrated on exploring the fundamental concepts and principles of bionic products. In Chinese literature, the term “bionic design” highlights drawing inspiration from nature for innovative product design, aligning with the English term “biomimetic synthesis.” The latter emphasizes creating new materials and compounds by imitating natural processes. Concurrently, “product design” in Chinese literature aims to enhance user experience and market competitiveness, paralleling the English focus on “design” and “fabrication,” both underscoring bionic applications in design and manufacturing processes.
2 Development stage: As research progresses, it increasingly targets specific applications and technologies. In Chinese literature, “industrial design” and “form bionics” have emerged as research hotspots, highlighting domestic scholars’ growing interest in applying bionic products to the industrial sector, particularly in product form and function design. In English literature, the rising frequency of keywords like “tissue engineering” and “calcium carbonate” signals a burgeoning interest in the application of bionic materials in biomedical engineering, especially within tissue engineering and biomineralization.
3 Maturity stage: During the maturity stage, research in both fields demonstrates trends of diversification and in-depth exploration. In Chinese literature, the frequent use of keywords such as “innovative design,” “green design,” and “sustainability” highlights concerns about biomimetic products’ role in fostering product innovation and environmental protection. Meanwhile, in English literature, the recurring keywords “molecular recognition,” “cascade reactions,” and “stereochemistry” suggest detailed discussions on the precise molecular design of biomimetic products and their complex reaction processes.

When comparing research trends in Chinese and English literature, notable differences and connections emerge. Chinese literature tends to emphasize the integration of bionic product design with technological applications, drawing inspiration from nature to enhance both functionality and aesthetic value. This focus is closely tied to China’s unique natural environment and design culture. For instance, the frequent appearance of keywords like “cultural and creative products” and “children’s furniture” in Chinese literature highlights the application of bionic products in the cultural and creative industries and among specific consumer groups. In contrast, English literature places greater emphasis on the materials science and biomedical applications of bionic products, such as “drug delivery” and “bone tissue engineering.” This focus reflects the long-term investment and leadership of Western countries in biotechnology and materials science.

### 2.4 Quantitative analysis

#### 2.4.1 Descriptive statistical analysis

In this study, descriptive statistics were employed to analyze the fundamental characteristics of the surveyed subjects. The results are presented in Table 5.

**Table 5:** Descriptive statistics table.

Variable	Category	Frequency	Percentage (%)	Cumulative (%)
Gender	Male	332	50.2	50.2
	Female	330	49.8	100.0
Age	21–30 years	227	34.3	34.3
	30–35 years	218	32.9	67.2
	35–40 years	217	32.8	100.0
Region	Mainland China	346	52.3	52.3
	Taiwan	316	47.7	100.0
Education Level	Below High School	125	18.9	18.9
	High School/Vocational	156	23.6	42.4
	College/University	118	17.8	60.3
	Master’s Degree	142	21.5	81.7
	Doctorate	121	18.3	100.0
Marital status	Previously Married	345	52.1	52.1
	Currently Unmarried	317	47.9	100.0
Monthly income (RMB)	Below 3,000	148	22.4	22.4
	3,000–10,000	144	21.8	44.1
	10,000–20,000	164	24.8	68.9
	Above 20,000	206	31.1	100.0
	Total	662	100.0	-

This study employs descriptive statistics to analyze the variables. As shown in the table below, the absolute values of kurtosis are under 10, and those of skewness are below 3. Consequently, each variable can be considered normally distributed. Table 6 presents the analysis of the questionnaire variables.

**Table 6:** Analysis of questionnaire variables.

Variable	*N*	Minimum	Maximum	Mean	Std. deviation	Skewness	Kurtosis
Youth green aesthetics	662	1.33	7.00	3.9765	1.61217	0.000	−1.452
Relative advantage	662	1.00	7.00	3.8927	1.64849	−0.031	−0.988
Compatibility	662	1.00	7.00	3.9945	1.60741	−0.011	−0.966
Observability	662	1.00	7.00	4.0302	1.92796	−0.082	−1.338
Motivation	662	1.00	7.00	4.1062	1.59423	−0.130	−1.012
Triggers	662	1.00	7.00	3.9673	1.62673	0.037	−0.975
Ability	662	1.00	7.00	3.9879	1.67402	0.081	−1.101
Evocation of natural imagery	662	1.00	7.00	4.0171	1.62216	−0.017	−1.018
Biomimetic experience	662	1.00	7.00	4.1662	1.66498	−0.167	−1.015
Emotional fulfillment	662	1.00	7.00	4.0327	1.66937	−0.013	−1.056
Willingness to adopt green	662	1.00	7.00	3.9597	0.93548	0.007	−0.237

#### 2.4.2 Reliability analysis

In this study, scale data were chosen for reliability and validity analysis, with Cronbach’s Alpha employed to assess data reliability. Typically, a Cronbach’s Alpha coefficient exceeding 0.7 signifies high reliability of the questionnaire, allowing for further in-depth analysis of relevant factors.

The following table demonstrates that the Cronbach’s Alpha coefficients for each dimension and the overall questionnaire in this study exceed 0.7. Additionally, the Corrected Item-Total Correlation (CITC) is above 0.4, and the Cronbach’s coefficients after item deletion are lower than those of the corresponding dimensions. This suggests the questionnaire’s overall reliability is high, and no items need to be removed. The reliability analysis is presented in Table 7.

**Table 7:** Reliability analysis.

Variable	Item	Deleted Item Mean	Deleted Item Variance	Corrected Item–Total Correlation	Cronbach’s α if Item Deleted α	Cronbach’s α	Composite α
Youth green aesthetics	A01	31.91	164.185	0.863	0.962	0.966	0.957
	A02	31.95	165.081	0.850	0.962		
	A03	31.99	164.638	0.859	0.962		
	A04	31.97	165.129	0.857	0.962		
	A05	31.98	164.234	0.858	0.962		
	A06	31.97	166.552	0.851	0.962		
	A07	32.00	164.156	0.858	0.962		
	A08	31.93	165.584	0.853	0.962		
	A09	32.02	165.876	0.849	0.962		
Relative advantage	B01	7.82	11.206	0.856	0.889	0.927	
	B02	7.79	11.251	0.853	0.892		
	B03	7.75	11.345	0.842	0.901		
Compatibility	B04	8.02	10.779	0.826	0.883	0.916	
	B05	7.94	10.726	0.830	0.879		
	B06	8.02	10.805	0.834	0.875		
Observability	B07	8.06	15.003	0.905	0.928	0.953	
	B08	8.04	15.244	0.900	0.932		
	B09	8.08	15.406	0.897	0.934		
Motivation	C01	8.26	10.838	0.825	0.890	0.918	
	C02	8.18	10.430	0.843	0.875		
	C03	8.20	10.478	0.836	0.881		
Triggers	C04	7.90	11.111	0.840	0.894	0.924	
	C05	7.97	10.784	0.840	0.894		
	C06	7.94	11.070	0.855	0.882		
Ability	C07	8.00	11.790	0.840	0.893	0.924	
	C08	7.91	11.558	0.849	0.886		
	C09	8.02	11.564	0.844	0.890		
Evocation of natural imagery	D01	7.98	10.826	0.838	0.880	0.918	
	D02	8.07	10.753	0.836	0.882		
	D03	8.05	11.285	0.831	0.886		
Biomimetic experience	D04	8.34	11.555	0.856	0.900	0.931	
	D05	8.32	11.539	0.851	0.904		
	D06	8.34	11.325	0.865	0.894		
Emotional Fulfillment	D07	8.04	11.537	0.862	0.893	0.930	
	D08	8.05	11.568	0.855	0.898		
	D09	8.11	11.515	0.850	0.902		
Willingness to adopt green packaging	E01	31.66	56.286	0.745	0.919	0.928	
	E02	31.66	56.278	0.739	0.919		
	E03	31.66	56.680	0.742	0.919		
	E04	31.68	55.745	0.755	0.918		
	E05	31.68	57.222	0.719	0.920		
	E06	31.70	57.351	0.711	0.921		
	E07	31.72	56.246	0.739	0.919		
	E08	31.66	56.327	0.732	0.920		
	E09	31.68	56.493	0.735	0.919		

#### 2.4.3 Validity analysis

In this study, factor analysis was utilized to assess validity. Typically, for validity analysis, a Kaiser-Meyer-Olkin (KMO) value exceeding 0.7 indicates that the questionnaire data is appropriate for factor analysis. As shown in the following table, all KMO test values surpass 0.7, and the significance value (Sig) for Bartlett’s test of sphericity is 0.000, demonstrating significant validity at the 0.001 level. Consequently, factor analysis is deemed appropriate. The results of the KMO and Bartlett’s tests are detailed in Table 8.

**Table 8:** KMO and Bartlett’s test.

Kaiser–Meyer–Olkin (KMO) measure of sampling adequacy		0.906
Bartlett’s test of sphericity	Statistic	25126.320
	df	990
	Sig.	0.000

An in-depth analysis reveals that the factors extracted from the scale explain a total variance of 65.494%, demonstrating strong explanatory power. The 11 extracted factors effectively preserve the original data’s information. Additionally, the extracted variance of the first factor loading, without rotation, is 25.784%, which is below 40%. This indicates that there is no significant common method bias in the questionnaire. The details are presented in Table 9.

**Table 9:** Total variance explained table.

Total variance explained									
Component	Initial eigenvalues			Extraction sums of squared loadings			Rotation sums of squared loadings		
	Total	% of Variance	Cumulative %	Total	% of Variance	Cumulative %	Total	% of Variance	Cumulative %
1	11.603	25.784	25.784	11.603	25.784	25.784	7.167	15.926	15.926
2	3.211	7.135	32.919	3.211	7.135	32.919	5.843	12.985	28.911
3	2.966	6.590	39.510	2.966	6.590	39.510	2.765	6.144	35.055
4	2.813	6.251	45.761	2.813	6.251	45.761	2.620	5.822	40.876
5	2.667	5.926	51.686	2.667	5.926	51.686	2.608	5.795	46.672
6	2.595	5.766	57.452	2.595	5.766	57.452	2.603	5.785	52.456
7	2.515	5.589	63.041	2.515	5.589	63.041	2.599	5.775	58.232
8	2.355	5.234	68.275	2.355	5.234	68.275	2.596	5.769	64.001
9	2.185	4.856	73.131	2.185	4.856	73.131	2.592	5.759	69.760
10	2.133	4.739	77.870	2.133	4.739	77.870	2.577	5.727	75.487
11	1.503	3.341	81.211	1.503	3.341	81.211	2.576	5.723	81.211
12	0.515	1.145	82.356						
13	0.462	1.027	83.383						
14	0.448	0.996	84.379						
15	0.423	0.939	85.318						
16	0.407	0.905	86.224						
17	0.401	0.890	87.114						
18	0.377	0.837	87.951						
19	0.342	0.759	88.710						
20	0.293	0.650	89.360						
21	0.291	0.647	90.007						
22	0.267	0.593	90.600						
23	0.260	0.578	91.178						
24	0.245	0.543	91.722						
25	0.242	0.537	92.259						
26	0.231	0.513	92.772						
27	0.223	0.496	93.269						
28	0.221	0.492	93.761						
29	0.220	0.490	94.251						
30	0.208	0.461	94.712						
31	0.203	0.451	95.163						
32	0.200	0.444	95.607						
33	0.191	0.425	96.032						
34	0.178	0.395	96.427						
35	0.174	0.387	96.814						
36	0.173	0.384	97.198						
37	0.166	0.368	97.566						
38	0.160	0.357	97.922						
39	0.153	0.341	98.263						
40	0.148	0.329	98.592						
41	0.145	0.322	98.914						
42	0.140	0.310	99.224						
43	0.128	0.285	99.509						
44	0.116	0.257	99.766						
45	0.105	0.234	100.000						

The factor loadings in the table demonstrate that all scale items align with their respective preset dimensions, confirming the questionnaire’s strong structural validity. Consequently, the data derived from the questionnaire is suitable for further analysis. Overall, as indicated in Table 10, the questionnaire exhibits high reliability and validity, making it a dependable and effective tool for research analysis.

**Table 10:** Rotated component matrix.

Rotated component matrix											
	Component 1										
	1	2	3	4	5	6	7	8	9	10	11
Youth green aesthetics 1	0.845										
Youth green aesthetics 2	0.832										
Youth green aesthetics 3	0.836										
Youth green aesthetics 4	0.830										
Youth green aesthetics 5	0.831										
Youth green aesthetics 6	0.852										
Youth green aesthetics 7	0.852										
Youth green aesthetics 8	0.834										
Youth green aesthetics 9	0.860										
Relative advantage 1					0.919						
Relative advantage 2					0.923						
Relative advantage 3					0.918						
Compatibility 1										0.907	
Compatibility2										0.917	
Compatibility 3										0.917	
Observability 1			0.953								
Observability 2			0.950								
Observability 3			0.953								
Motivation 1											0.911
Motivation2											0.919
Motivation 3											0.912
Trigger 1							0.908				
Trigger 2							0.916				
Trigger 3							0.929				
Ability 1									0.913		
Ability 2									0.918		
Ability 3									0.912		
Natural Imagery arousal 1								0.914			
Natural imagery arousal 2								0.919			
Natural imagery arousal 3								0.912			
Biomorphic experience 1						0.906					
Biomorphic experience 2						0.914					
Biomorphic experience 3						0.908					
Emotional fulfillment 1				0.920							
Emotional fulfillment 2				0.912							
Emotional fulfillment 3				0.922							
Green packaging adoption intention 1		0.757									
Green packaging adoption intention 2		0.729									
Green packaging adoption intention 3		0.746									
Green packaging adoption intention 4		0.734									
Green packaging adoption intention 5		0.709									
Green packaging adoption intention 6		0.741									
Green packaging adoption intention 7		0.737									
Green packaging adoption intention 8		0.713									
Green packaging adoption intention 9		0.743									

#### 2.4.4 Validity analysis

This study utilized Pearson correlation analysis to examine significant correlations among various variables. The results of this analysis are displayed in Table 11.

1 The factors most relevant to the “adoption willingness”:

**Table 11:** Correlation analysis.

Variables	YGA	RA	COMP	OBS	MOT	TRIG	ABIL	NIA	BIO	EF	GPAI
Youth green aesthetics (YGA)	1										
Relative advantage (RA)	0.138^*^	1									
Compatibility (COMP)	0.151^*^	0.036	1								
Observability (OBS)	−0.071	−0.042	−0.101^*^	1							
Motivation (MOT)	0.131^*^	0.013	−0.045	−0.003	1						
Trigger (TRIG)	0.162^*^	0.035	−0.020	0.054	0.009	1					
Ability (ABIL)	0.194^*^	0.061	0.052	−0.070	−0.015	0.027	1				
Natural imagery arousal (NIA)	0.161^*^	0.010	0.025	−0.045	0.031	0.046	−0.004	1			
Biomorphic experience (BIO)	0.289^*^	−0.020	0.095^*^	−0.090^*^	0.055	0.044	0.108^*^	0.091^*^	1		
Emotional fulfillment (EF)	0.197^*^	−0.007	0.073	0.008	0.052	0.039	−0.058	0.016	0.085^*^	1	
Green packaging adoption intention (GPAI)	0.591^*^	0.240^*^	0.188^*^	−0.029	0.230^*^	0.238^*^	0.238^*^	0.217^*^	0.281^*^	0.252^*^	1

*Indicates a significant correlation at the 0.01 level (two-tailed).

Green packaging (correlation coefficient: 0.591)

Description: This factor has the strongest correlation with “adoption intention” among all variables. It suggests that young consumers’ positive perceptions and attitudes toward “green packaging” are the primary drivers of their purchase intentions.

2 Factors significantly correlated with the “willingness to adopt”:

Multiple factors showed a moderate positive correlation with the intention to adopt, including:

Relative advantage (0.138): The greater the perceived benefits, such as increased user-friendliness or fashionability, of green packaging, the stronger the purchase intention.

Compatibility (0.151): The more closely green packaging aligns with the values and lifestyles of young people, the stronger their intention to purchase.

Motivation (0.230): An individual’s intrinsic motivation for environmental protection significantly enhances their intention to make a purchase.

Triggers (0.238): External factors, like advertisements and recommendations, can effectively stimulate purchase intentions.

Ability (0.238): The belief in one’s ability, encompassing both affordability and convenience of purchase, significantly influences willingness.

Natural imagery (0.217): Packaging that conveys natural and pure imagery can enhance purchase intention.

Bionic experience (0.281): The natural associations and experiences evoked by packaging design can significantly enhance purchasing behavior.

Emotional satisfaction (0.252) refers to the positive emotions, such as peace of mind and pleasure, that arise from using green packaging, which can, in turn, boost willingness.

3 Factors that have no or a weak relationship with “adoption willingness”:

Observability (−0.029): The correlation coefficient is nearly and not significant, suggesting that there is virtually no relationship between the visibility and conspicuousness of the packaging and the purchase intentions of young people.

#### 2.4.5 Confirmatory factor analysis

**Figure 5:**
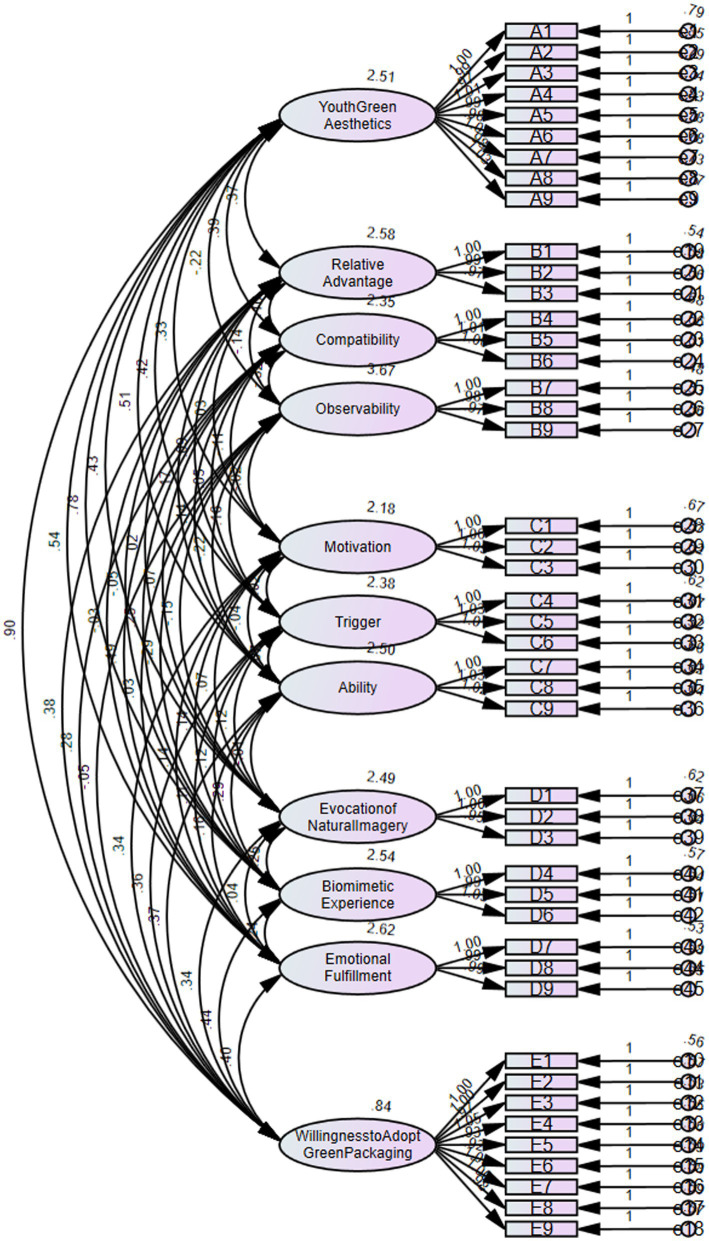
Measurement model diagram.

The standardized factor loadings for each item, along with the CR and AVE values for each dimension, meet the established standards (Figure 5). This indicates that the data exhibit strong composite reliability and structural validity. Details on composite reliability and structural validity are presented in Table 12.

**Table 12:** Composite reliability and construct validity.

Construct	Item	Standardized Factor Loading	CR	AVE	√AVE
Youth green aesthetics	A15	0.872	0.957	0.759	0.871
	A14	0.862			
	A13	0.867			
	A12	0.881			
	A11	0.866			
	A10	0.871			
	A09	0.879			
Relative advantage	B01	0.910	0.927	0.809	0.899
	B02	0.901			
	B03	0.887			
Compatibility	B04	0.881	0.916	0.784	0.885
	B05	0.885			
	B06	0.891			
Observability	B07	0.940	0.953	0.871	0.933
	B08	0.933			
	B09	0.927			
Motivation	C01	0.875	0.919	0.790	0.889
	C02	0.900			
	C03	0.891			
Trigger	C04	0.890	0.924	0.802	0.896
	C05	0.889			
	C06	0.908			
Ability	C07	0.889	0.924	0.801	0.895
	C08	0.900			
	C09	0.896			
Natural imagery arousal	D01	0.894	0.919	0.790	0.889
	D01	0.890			
	D03	0.883			
Biomorphic experience	D04	0.903	0.931	0.817	0.904
	D05	0.893			
	D06	0.916			
Emotional fulfillment	D07	0.912	0.930	0.815	0.903
	D08	0.904			
	D09	0.892			
Green packaging adoption intention	E01	0.775	0.928	0.588	0.767
	E02	0.773			
	E03	0.773			
	E04	0.790			
	E05	0.751			
	E06	0.737			
	E07	0.770			
	E08	0.766			

#### 2.4.6 Discriminant validity

In this study, we compared the square roots of the average variance extracted (AVE) for each dimension with the correlation coefficients between dimensions. The results indicate that the square root of the AVE for each dimension exceeds the inter-dimensional correlations, demonstrating that internal correlations within each dimension are stronger than those between dimensions. This finding suggests that the data exhibits strong discriminant validity. In summary, the data demonstrates good reliability and validity, making it suitable for further analysis. Discriminant validity is detailed in Table 13.

**Table 13:** Discriminant validity.

Variables	YGA	RA	COMP	OBS	MOT	TRIG	ABIL	NIA	BIO	EF	GPAI
Youth green aesthetics (YGA)	0.871										
Relative advantage (RA)	0.145	0.899									
Compatibility (COMP)	0.161	0.042	0.885								
Observability (OBS)	−0.108	−0.074	−0.045	0.933							
Motivation (MOT)	0.139	−0.049	−0.007	0.012	0.889						
Trigger (TRIG)	0.170	0.011	0.036	−0.021	0.056	0.896					
Ability (ABIL)	0.205	0.066	0.056	−0.073	−0.016	0.029	0.895				
Natural imagery arousal (NIA)	0.171	0.010	0.027	−0.048	0.032	0.049	−0.004	0.889			
Biomorphic experience (BIO)	0.307	0.061	−0.096	0.104	−0.020	0.047	0.116	0.098	0.904		
Emotional fulfillment (EF)	0.211	−0.011	0.078	0.009	0.057	0.043	−0.061	0.017	0.092	0.903	
Green packaging adoption intention (GPAI)	0.624	0.272	0.303	0.237	0.259	0.257	0.249	−0.031	0.203	0.259	0.767

#### 2.4.7 Mediating effect

**Figure 6:**
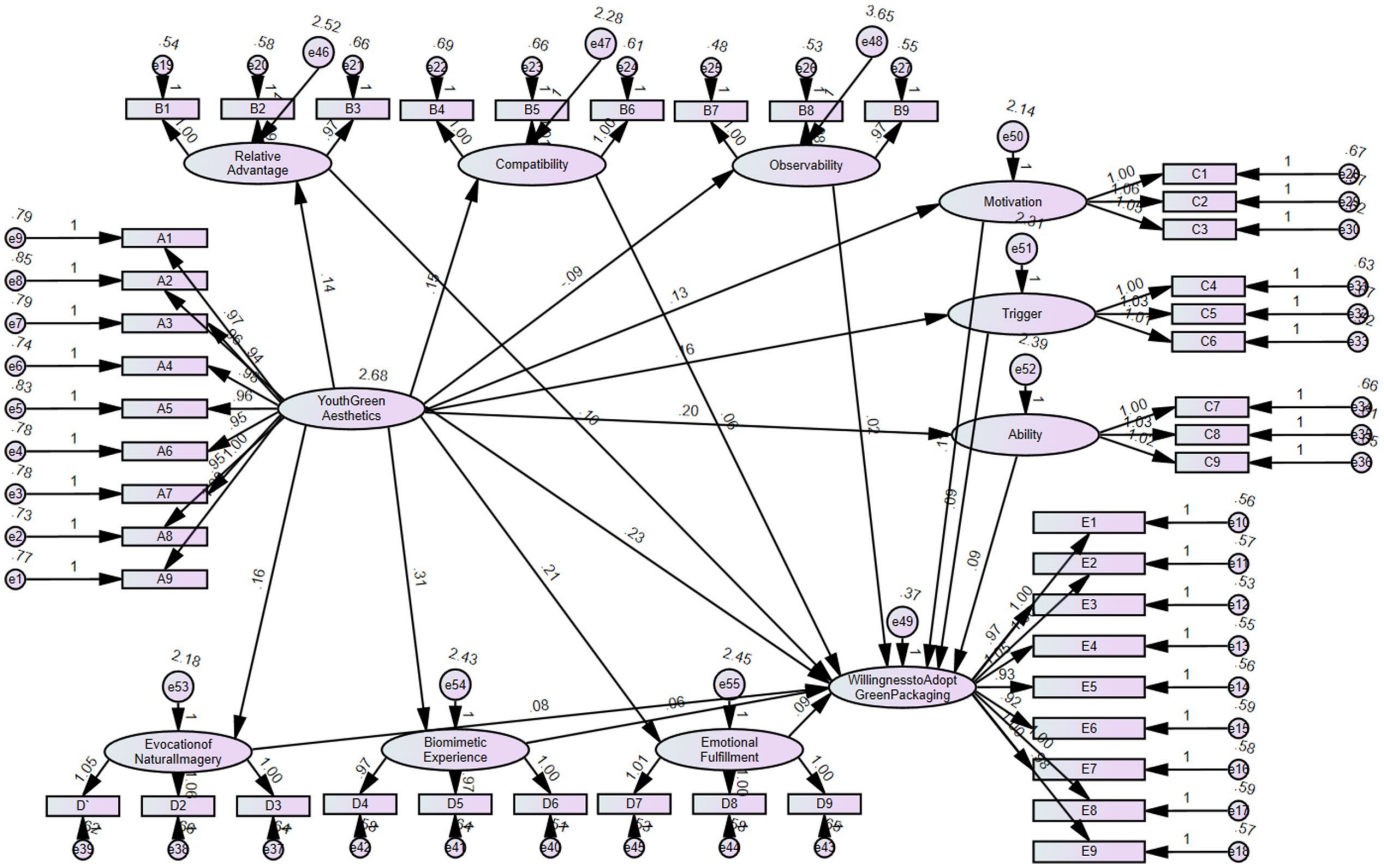
Structural model diagram.

In this study, we employed the Bootstrap analysis method to verify the mediating effect of the relevant hypotheses. With a Bootstrap sample size of 200, we conducted the mediation effect test at a 95% confidence level. Figure 6 illustrates the structural model diagram, while Table 14 presents the mediation effect.

1 Established mediating paths (8 in total)

A H2a path: Youth green aesthetics → Relative advantage → Willingness to purchase green packaging

**Table 14:** Mediating effects.

Hypothesis	Parameter	Estimate	Lower	Upper	P	Conclusion
H2a	Youth green aesthetics → Relative advantage → Green packaging adoption intention	0.026	0.013	0.040	0.001	Supported
H2b	Youth green aesthetics → Compatibility → Green packaging adoption intention	0.018	0.008	0.028	0.001	Supported
H2c	Youth green aesthetics → Observability → Green packaging adoption intention	−0.003	−0.008	0.001	0.250	Not supported
H3a	Youth green aesthetics → Motivation → Green packaging adoption intention	0.024	0.012	0.038	0.002	Supported
H3b	Youth green aesthetics → Trigger → Green packaging adoption intention	0.026	0.015	0.040	0.001	Supported
H3c	Youth green aesthetics → Ability → Green packaging adoption intention	0.031	0.018	0.046	0.001	Supported
H4a	Youth green aesthetics → Natural imagery arousal → Green packaging adoption intention	0.023	0.012	0.037	0.001	Supported
H4b	Youth green aesthetics → Biomorphic experience → Green packaging adoption intention	0.034	0.017	0.051	0.002	Supported
H4c	Youth green aesthetics → Emotional fulfillment → Green packaging adoption intention	0.033	0.019	0.049	0.001	Supported

The mediation effect value is 0.026.

The 95% confidence interval [0.013, 0.040] does not include 0.

*p* = 0.001 < 0.01, indicating that the path is significantly valid.

B H2b path: Youth green aesthetics → Compatibility → Willingness to purchase green packaging

The mediation effect value is 0.018.

The 95% confidence interval [0.008, 0.028] does not include 0.

*p* = 0.001 < 0.01, indicating that the path is significantly valid.

C H3a path: Youth’s green aesthetic perception → Motivation → Purchase intention of green packaging

The mediating effect value is 0.024.

The 95% confidence interval [0.012, 0.058] does not include 0.

*p* = 0.002 < 0.01, indicating that the path is significantly valid.

D H3b path: Youth green aesthetics → Trigger → Purchase intention of green packaging

The mediating effect value is 0.026.

The 95% confidence interval [0.015, 0.040] does not contain 0.

*p* = 0.001 < 0.01, indicating that the path is significantly valid.

E H3c path: Youth green aesthetics → Ability → Willingness to purchase green packaging

The mediation effect value is 0.031.

The 95% confidence interval [0.018, 0.046] does not include 0.

*p* = 0.001 < 0.01, indicating that the path is significantly valid.

F H4a Path: Youth’s perception of green aesthetics → Evocation of green imagery → Intention to purchase green packaging

The mediating effect value is 0.023.

The 95% confidence interval [0.012, 0.037] does not include 0.

*p* = 0.001 < 0.01, indicating that the path is significantly valid.

G H4b path: Youth’s green aesthetic perception → Bionic experience → Purchase intention of green packaging

The mediating effect value is 0.034.

The 95% confidence interval [0.017, 0.051] does not include 0.

*p* = 0.002 < 0.01, indicating that the path is significantly established.

H H4c path: Youth green aesthetics → Emotional satisfaction → Purchase intention for green packaging

The mediating effect value is 0.033.

The 95% confidence interval [0.019, 0.049] does not include 0.

*p* = 0.001 < 0.01, indicating that the path is significantly valid.

2 Non-established mediation paths (1 path)

H2c path: Youth green aesthetics → Observability → Willingness to purchase green packaging.

The mediating effect value is −0.003.

The 95% confidence interval [−0.008, 0.001] contains 0.

*p* = 0.250 > 0.05, indicating that the path is not significant.

## 3 Discussion

The discussion of this study is summarized in six points as follows:

1 The diffusion effect of youth green aesthetics in social media scenarios

The findings indicate that youth green aesthetics exert a significant influence on the intention to adopt green packaging, primarily through perceived relative advantage and compatibility. Rather than treating green aesthetics as a purely visual preference, the results suggest that it functions as a value-laden cognitive schema that shapes how young consumers interpret innovation attributes within social media environments. In this sense, aesthetics operate as an upstream framing mechanism that conditions subsequent evaluative judgments, rather than as an isolated design attribute.

Importantly, this diffusion process should not be interpreted as a linear transmission from exposure to adoption. Social media platforms introduce algorithmic amplification and peer-based filtering, which may selectively reinforce content that already aligns with users’ pre-existing environmental identities. Consequently, the observed diffusion effect may partly reflect value homophily and selective exposure, rather than pure innovation-driven persuasion (Maidin et al., 2024). This interpretation cautions against overstating the causal power of aesthetic diffusion alone and aligns with recent critiques that emphasize the co-evolution of individual preferences and platform logics in sustainable consumption contexts.

Furthermore, the diffusion observed in this study is confined to intention formation, not verified behavioral adoption. While green aesthetics appear to lower psychological resistance to green packaging, the transition from intention to consistent behavior remains contingent on situational constraints, economic considerations, and institutional infrastructures. Therefore, claims regarding large-scale behavioral transformation should be treated with caution (Bozkurt et al., 2014).

2 The mediating role of persuasive design in attitude–behavior transformation

The mediating effects of motivation, ability, and trigger provide empirical support for the relevance of persuasive design mechanisms in shaping adoption intentions. However, rather than confirming a deterministic “aesthetics → behavior” pathway, the results suggest that persuasive design functions as a conditional facilitator that translates aesthetic appreciation into actionable intention under favorable circumstances.

Among the three components, ability and trigger exhibit relatively stronger mediating effects, indicating that perceived feasibility and situational prompts may be more decisive than motivational arousal alone. This finding nuances the Fogg Behavior Model by suggesting that, in sustainability-related contexts, motivation may already be relatively high among environmentally aware youth, while behavioral conversion depends more on reduced friction and timely cues (Ikejima et al., 2025; O'Shea et al., 2022).

At the same time, the persuasive effects identified here should be interpreted with caution due to potential social desirability bias. Given the pro-environmental framing of questionnaire items, respondents may overreport motivational alignment or responsiveness to persuasive cues. Thus, the mediating role of persuasive design reflects perceived behavioral readiness rather than observed action, underscoring the need for experimental or longitudinal validation in future studies.

3 Emotional and ethical value gains brought by biophilic design

The significant mediating roles of natural imagery, biomimetic experience, and emotional fulfillment highlight the importance of affective and ethical dimensions in green packaging adoption. These results support biophilia theory by demonstrating that natural cues do more than enhance aesthetic pleasure; they contribute to moral resonance and emotional reassurance, which strengthen adoption intentions.

However, these emotional gains should not be equated with stable ethical commitment. The emotional responses elicited by biophilic design may be context-dependent and transient, particularly within fast-paced social media environments characterized by novelty saturation. In such contexts, emotional fulfillment may reinforce short-term positive evaluation without necessarily leading to long-term behavioral consistency (Wang et al., 2025).

Moreover, cultural specificity must be considered. The symbolic meanings attached to natural imagery and biomimetic forms may differ between Mainland China and Taiwan due to divergent cultural narratives surrounding nature, tradition, and modernity. As such, the emotional and ethical pathways identified in this study should be understood as contextually embedded, rather than universally generalizable mechanisms (Chen et al., 2022).

4 Weakening and differential explanations of observability effects

Contrary to classical diffusion theory, observability does not exhibit a significant mediating effect. Rather than interpreting this as theoretical inconsistency, the result reflects a structural shift in visibility regimes under social media conditions. When green packaging visuals become ubiquitous, visibility alone loses discriminative power, leading to diminishing marginal effects.

This finding suggests that observability has transitioned from a scarcity-based advantage to a baseline condition. What differentiates adoption intention is no longer whether green packaging is visible, but whether it conveys meaningful narratives, interactional depth, or identity alignment (Menges, 2012). In this sense, observability may function as a necessary but insufficient condition, overshadowed by more interpretive and experiential factors.

Additionally, the non-significant result may stem from measurement limitations, as observability was operationalized primarily through self-reported perceptual items. Future research employing behavioral metrics (e.g., actual sharing or display behaviors) may provide a more nuanced understanding of observability effects (Lenas et al., 2009; Yang and Tang, 2020; Chang, 2023).

5 Policies, social norms, and future development directions

The findings indicate that youth adoption intentions are shaped by a multi-level interplay between individual aesthetics, persuasive mechanisms, and broader institutional signals. Policies such as carbon labeling and plastic reduction mandates appear to function less as direct motivators and more as legitimizing frameworks that reduce skepticism and greenwashing concerns.

Social norms emerging from online communities further reinforce these effects by normalizing green packaging as an expected rather than exceptional choice. However, normative pressure alone may also generate compliance-oriented intentions that lack deep personal commitment. This duality highlights the importance of aligning policy instruments, platform governance, and design strategies to foster not only adoption intention but also sustained engagement (Yue et al., 2024).

Future development should therefore move beyond isolated design optimization toward systemic coordination among policymakers, platforms, and designers. Longitudinal tracking, cross-cultural comparison, and experimental validation are essential to clarify whether the observed intention-based mechanisms can translate into durable behavioral change (Nowak and Fic, 2011).

6 Research summary

One noteworthy finding of this study is that observability did not exhibit a significant mediating effect between youth green aesthetics and the willingness to adopt green packaging, despite its theoretical importance within Rogers’ diffusion of innovations framework. According to diffusion theory, observability refers to the degree to which the results of an innovation are visible to others, which generally facilitates imitation and accelerates diffusion processes.

However, the empirical results of this study suggest that in the context of biomimetic cultural and creative packaging within social media environments, observability may play a relatively weaker role compared with other mechanisms. Several theoretical explanations may account for this outcome.

First, in contemporary social media ecosystems, visual exposure is already highly saturated. Platforms such as short-video applications, influencer marketing channels, and image-based sharing communities continuously expose users to various product aesthetics. As a result, the mere visibility of green packaging may no longer serve as a distinctive stimulus influencing adoption decisions. Instead, users may rely more on perceived experiential and emotional attributes, such as biomimetic experience and emotional fulfillment, which were found to have stronger mediating effects in this study.

Second, the adoption of green packaging among young consumers appears to be driven more strongly by value alignment and identity expression than by simple visibility. In the context of youth green aesthetics, individuals tend to interpret sustainable packaging as a symbol of environmental consciousness and lifestyle identity. Therefore, constructs such as compatibility and perceived ability, which reflect alignment with personal values and ease of behavioral implementation, exert stronger explanatory power than observability.

Third, the results may also reflect the shift from passive observation to interactive engagement in digital environments. Unlike traditional diffusion contexts where innovations spread through observable usage, social media interactions involve narrative framing, community endorsement, and algorithmic amplification. In such environments, persuasive triggers and experiential narratives may play a more critical role in shaping behavioral intention than simple visual exposure.

Taken together, these findings suggest that while observability remains a theoretically relevant construct in diffusion theory, its influence may diminish in high-visual, highly mediated social media environments where aesthetic saturation reduces the marginal impact of visibility. Instead, mechanisms related to experiential engagement, emotional resonance, and behavioral facilitation appear to be more influential in transforming youth green aesthetics into sustainable consumption behavior.

This result also echoes recent studies indicating that contemporary green consumption is increasingly driven by identity signaling, emotional engagement, and social narrative construction, rather than by purely informational visibility of sustainable products.

### 3.1 Research limitations and suggestions

1 The study focused on Chinese youths aged 18 and above, utilizing an online questionnaire with non-random sampling. Additionally, the expert interviews involved only experts and scholars from within China, without input from foreign scholars.
2 In the future, the structural model of this study could include moderating effects to boost its explanatory power, thereby improving the model. This enhancement is expected to further increase the quality and reliability of research on natural information commodities.
3 This study did not concentrate on specific ethnic groups or regions. Future research should adopt a geographical perspective to examine regional consumer differences. This approach will aid in developing tailored business strategies or models for various areas, facilitating the genuine implementation and expansion of sustainable consumption behavior across different regions.
4 This study has several methodological limitations. First, the qualitative phase relied on a small number of expert interviews, which functioned primarily as conceptual guidance rather than a fully saturated grounded theory analysis; systematic coding and category development were therefore limited. Second, the survey employed non-random online sampling, which constrains external validity. The operational definition of “youth” extended to respondents aged 40, which may weaken construct precision. Third, newly developed measurement items were validated within a single sample without external scale validation. Finally, common method bias was assessed using Harman’s single-factor test only. Future research should adopt multi-source data, stricter age definitions, longitudinal designs, and advanced bias-control techniques.
5 Several limitations should be noted regarding data analysis and interpretation. Although the SEM results demonstrate acceptable reliability and model fit, the analysis was primarily statistical and provided limited theoretical interpretation of path coefficients. Some constructs with relatively weak associations with adoption intention were retained in the model, and their theoretical relevance warrants further clarification. In particular, the non-significant role of observability, while empirically robust, was not fully theorized within alternative diffusion contexts.

Despite the theoretical and empirical contributions of this study, several limitations should be acknowledged in order to provide a balanced interpretation of the findings and to indicate directions for future research.

First, the present study adopts a cross-sectional survey design, which captures respondents’ perceptions and intentions at a single point in time. Although structural equation modeling enables the examination of theoretically grounded relationships among variables, the cross-sectional nature of the data limits the ability to establish causal relationships. Future research could adopt longitudinal or experimental designs to track changes in consumer perceptions and behaviors over time. For example, longitudinal panel data could reveal how youth green aesthetic perceptions evolve alongside exposure to social media content and sustainability campaigns, thereby providing stronger evidence regarding causal mechanisms.

Second, the study focuses on adoption intention rather than actual behavioral outcomes. While behavioral intention is widely recognized in consumer research as a strong predictor of actual behavior, intention does not always translate into real-world purchasing or usage behavior due to situational constraints, price considerations, or contextual factors. Future research could incorporate behavioral tracking methods, purchase records, or experimental simulations to examine the gap between intention and behavior, thereby providing a more comprehensive understanding of green packaging adoption.

Third, the use of self-reported questionnaire data may introduce potential biases, including social desirability bias and common method bias. In studies related to environmentally responsible behavior, respondents may tend to overreport environmentally friendly attitudes or intentions in order to align with socially desirable norms. Although statistical procedures such as Harman’s single-factor test were applied to assess common method variance and the results did not indicate severe bias, future research could combine multiple data sources—such as behavioral observation, experimental tasks, or digital trace data—to further improve measurement robustness.

Fourth, the sampling approach used in this study is based on non-probability online sampling, which may limit the generalizability of the findings. Although the sample includes respondents from multiple social media communities across Mainland China and Taiwan and covers a relatively diverse demographic background, the participants are still drawn from self-selected online user groups. Future studies may adopt probability sampling methods or expand data collection to broader population groups in order to enhance external validity.

Finally, the geographical scope of the study is limited to Mainland China and Taiwan. Cultural values, environmental awareness, and social media ecosystems may differ substantially across regions, which means that the findings may not be fully generalizable to other cultural contexts. Future research could conduct cross-cultural comparative studies involving additional countries or regions to examine whether the mechanisms linking green aesthetics, persuasive design, and biophilic responses operate similarly across different cultural environments.

Despite these limitations, the present study provides an important step toward understanding how youth green aesthetics influence sustainable packaging adoption within social media environments. By integrating innovation diffusion theory, persuasive design theory, and biophilia theory, the research offers a multi-level explanation of how aesthetic perception, behavioral facilitation, and emotional engagement jointly shape sustainable consumption decisions.

## 4 Conclusion

This study integrates innovation diffusion theory, persuasive design theory, and biophilia theory to examine the psychological mechanisms and behavioral pathways underlying young consumers’ adoption of green packaging for biomimetic cultural and creative products within social media environments. By combining expert interviews with structural equation modeling (SEM), the study provides empirical evidence that youth green aesthetics function as a central value orientation shaping green packaging adoption both directly and indirectly.

The results indicate that youth green aesthetics exert a strong direct effect on adoption intention and operate through three distinct but interrelated mediating mechanisms: perceived innovation attributes (relative advantage and compatibility), persuasive design mechanisms (motivation, ability, and trigger), and biophilic responses (natural imagery, biomimetic experience, and emotional fulfillment). Notably, observability does not significantly mediate adoption intention, suggesting that in highly visualized social media contexts, mere visibility of green packaging is insufficient. Instead, internalized value alignment, emotional resonance, and behavioral facilitation play a more decisive role in shaping adoption behavior.

From a theoretical perspective, this study contributes to the green packaging and sustainable consumption literature by moving beyond a traditional function–performance paradigm. Rather than treating aesthetics as a peripheral design attribute, the findings demonstrate that green aesthetics operate as a psychological and symbolic driver that links diffusion processes, persuasive mechanisms, and nature-based emotional responses. The proposed cross-level framework of “aesthetics–diffusion–persuasion–biophilia” clarifies the complementary roles of these theories and offers a more integrated explanation of how aesthetic perception is translated into sustained pro-environmental behavior in digital contexts.

From a practical standpoint, the findings suggest several implications. First, brands should conceptualize green aesthetics as a strategic resource rather than a decorative element, leveraging biomimetic forms, natural symbols, and material narratives to foster emotional and ethical identification. Second, social media strategies should focus on strengthening the “aesthetics-to-action” conversion by embedding persuasive design elements—such as interactive triggers, ritualized unboxing experiences, and low-effort recycling cues—that reduce behavioral friction. Third, policymakers and platform providers can support sustainable consumption by aligning regulatory standards, certification labels, and algorithmic visibility with credible green narratives, thereby reinforcing collective norms around environmentally responsible consumption.

Despite these contributions, several limitations must be acknowledged. First, the study employs a cross-sectional research design, which restricts causal inference and does not capture the temporal evolution of youth green aesthetics or adoption behavior. Second, the qualitative phase relied on a limited number of expert interviews, which served primarily as conceptual guidance rather than a fully saturated qualitative analysis. Third, the survey used non-random online sampling within a specific cultural context, potentially limiting generalizability. Finally, although multiple mediating mechanisms were identified, the study did not experimentally manipulate persuasive or biophilic design elements, constraining conclusions regarding their causal efficacy.

Future research should address these limitations through longitudinal designs that track changes in green aesthetic values and adoption behavior over time, as well as experimental studies that isolate and validate specific persuasive and biophilic mechanisms. In addition, cross-cultural replication across different social media ecosystems and cultural contexts would help determine the robustness and boundary conditions of the proposed framework. Such extensions would not only strengthen causal claims but also contribute to a more globally grounded understanding of youth-driven green consumption and sustainable design practices.

## Data Availability

The datasets presented in this study can be found in online repositories. The names of the repository/repositories and accession number(s) can be found in the article/supplementary material.
